# Obesity Differentially Affects Phenotypes of Polycystic Ovary Syndrome

**DOI:** 10.1155/2012/317241

**Published:** 2012-07-08

**Authors:** Carlos Moran, Monica Arriaga, Gustavo Rodriguez, Segundo Moran

**Affiliations:** ^1^Direction of Health Research and Training, Medical Unit of High Specialty, Gynecology and Obstetrics Hospital No. 4 Luis Castelazo Ayala, Mexican Institute of Social Security, Mexico City, DF 01090, Mexico; ^2^Health Research Council, Mexican Institute of Social Security, Mexico City, DF 06725, Mexico; ^3^General Hospital of Zone No. 8, Mexican Institute of Social Security, Mexico City, DF 01090, Mexico

## Abstract

Obesity or overweight affect most of patients with polycystic ovary syndrome (PCOS). Phenotypes are the clinical characteristics produced by the interaction of heredity and environment in a disease or syndrome. Phenotypes of PCOS have been described on the presence of clinical hyperandrogenism, oligoovulation and polycystic ovaries. The insulin resistance is present in the majority of patients with obesity and/or PCOS and it is more frequent and of greater magnitude in obese than in non obese PCOS patients. Levels of sexual hormone binding globulin are decreased, and levels of free androgens are increased in obese PCOS patients. Weight loss treatment is important for overweight or obese PCOS patients, but not necessary for normal weight PCOS patients, who only need to avoid increasing their body weight. Obesity decreases or delays several infertility treatments. The differences in the hormonal and metabolic profile, as well as the different focus and response to treatment between obese and non obese PCOS patients suggest that obesity has to be considered as a characteristic for classification of PCOS phenotypes.

## 1. Introduction 

Polycystic ovary syndrome (PCOS) is an endocrine and metabolic heterogeneous disorder, with a likely genetic origin [1–4], influenced by environmental factors [5, 6]. The main clinical features of PCOS are related to hyperandrogenism, such as hirsutism and menstrual disorders [7–9]. PCOS is also associated with overweight or obesity [8]. The etiology of PCOS is unknown. 

Obesity plays an important role in the pathogenesis of PCOS, and the majority of patients with PCOS are overweight or obese. However, these disorders are not considered as diagnostic criteria for PCOS, because not all obese women present hyperandrogenism. The purpose of this paper is to propose that the classification of PCOS phenotypes must take into consideration the presence of obesity because phenotypes are defined as all clinical characteristics produced by the interaction of heredity and environment in a disease or syndrome. 

## 2. Diagnosis 

The major criteria of PCOS, proposed in the consensus of the National Institutes of Health (NIH) in Bethesda, MD, USA, were as follows (in order of importance): (a) hyperandrogenism and/or hyperandrogenemia, (b) oligoovulation, (c) exclusion of other known disorders, and (d) possibly the characteristic morphology of polycystic ovaries on ultrasound [10]. At the Rotterdam PCOS Consensus Workshop Group, the presence of two out of the three following criteria was deemed necessary for diagnosis of PCOS: (a) oligoovulation or anovulation, (b) clinical and/or biochemical signs of hyperandrogenism, and (c) polycystic ovaries by ultrasound, also the exclusion of other related disorders [11]. The Androgen Excess and PCOS Society (AES-PCOS) considers with the following as PCOS: hyperandrogenism (hirsutism and/or hyperandrogenemia), ovarian dysfunction (oligo-anovulation and/or polycystic ovaries), and the exclusion of other androgen excess or related disorders [12]. 

## 3. Phenotypes

Certain single-nucleotide polymorphisms associated with obesity contribute to elevated body mass index (BMI) in PCOS, supporting the concept that its phenotypes are a consequence of a polygenic mechanism [13]. However, obesity is not taken into account for PCOS phenotypes. 

Phenotypes of PCOS patients can be classified as follows: (A) hyperandrogenism, oligoanovulation and polycystic ovaries by ultrasound; (B) hyperandrogenism and oligoanovulation (and normal appearance of the ovaries by ultrasound); (C) hyperandrogenism and polycystic ovaries by ultrasound (with regular ovulatory menstrual cycles); and (D) oligoanovulation, and polycystic ovaries by ultrasound (without hyperandrogenism). 

The NIH criteria recognize A and B phenotypes [10].**  **The Rotterdam consensus accepts all four phenotypes [11].**  **The AES-PCOS admits A, B, and C phenotypes [12].  Table 1 shows the frequency of each one of the phenotypes on different populations. Phenotype A is the most frequent (44–65%), followed by phenotype B (8–33%), then phenotype C (3–29%), and finally phenotype D (0–23%) [14–17].  Table 2 indicates the present proposal of phenotypes taking into account obesity. 

## 4. Prevalence of PCOS and/or Obesity

Polycystic ovary syndrome (PCOS) affects 4–7% of women in reproductive age [18–21]. It is considered one of the most frequent endocrine disorders in women of reproductive age [18]. It is noteworthy that PCOS explains 55–80% of the patients with hyperandrogenism (Table 3) [7–9]. Overweight or obesity affects approximately 60–80% of PCOS patients [8].

## 5. Clinical Presentation in Obese and Non Obese PCOS Patients

It has been reported that obese PCOS patients have a greater prevalence of some clinical manifestations, such as hirsutism and menstrual disorders, than non obese PCOS patients [22]; however, other studies have not found such differences [23]. The discrepancies between these studies may be the result of different diagnostic criteria used to classify obesity and PCOS.

## 6. Role of Obesity in the Pathophysiology of PCOS 

The main pathophysiological components of PCOS are gonadotropic dysfunction and insulin resistance [24–26]. It has been found that both of these components are related to the BMI.

### 6.1. Gonadotropic Dysfunction

In some studies, it has been observed that dissociation of luteinizing hormone (LH) to follicle-stimulating hormone (FSH) is higher in PCOS patients with normal weight than in obese PCOS patients [25] although this observation has not been found in other studies [24, 26] (Table 4). 

### 6.2. Insulin Resistance

**Figure 1:**
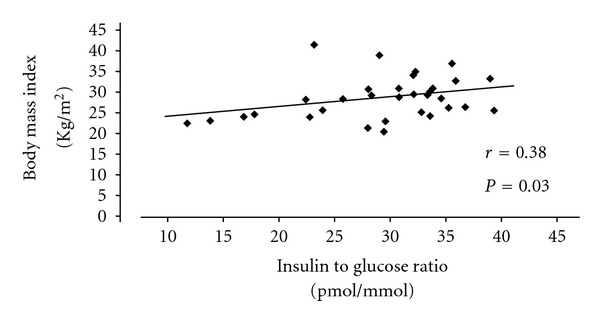
Relationship between body mass and insulin resistance in patients with polycystic ovary syndrome.There is a significantly positive correlation between body mass index and insulin to glucose ratio. Modified from Moran et al. [24].

PCOS is associated to metabolic disorders like insulin resistance [27–30], becoming a risk factor for the development of carbohydrate intolerance and type 2 diabetes mellitus [31, 32]. Insulin resistance appears in women with PCOS with suitable weight [27], and overweight or obesity [24], but it is more frequent and of greater magnitude when there is obesity [24, 33]. The insulin resistance is approximately twofold that of nonobese PCOS patients (Table 4). The magnitude of overweight and obesity is directly related to insulin resistance in PCOS patients (Figure 1) [24]. 

## 7. Body Fat Distribution 

**Figure 2:**
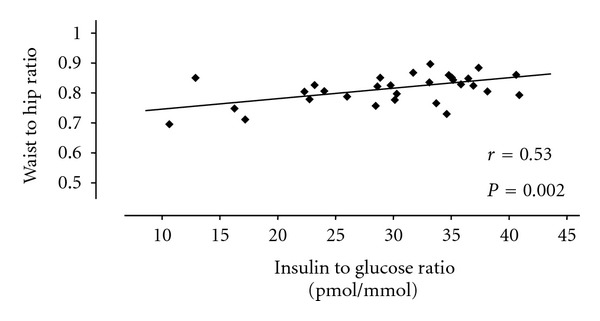
Relationship between body fat distribution and insulin resistance in patients with polycystic ovary syndrome. A significantly positive correlation between waist to hip ratio and insulin to glucose ratio is observed. Modified from Moran et al. [24].

The type of obesity is predominantly of abdominal distribution in PCOS patients [18, 24]. The upper body adiposity is related to insulin resistance in PCOS patients (Figure 2) [24]. In addition, upper body adiposity has been found to be associated with a higher percentage of anovulation in comparison to lower body adiposity (83% versus 65%, resp.) [34].

## 8. Ovarian Morphology of Polycystic Ovaries

There is some evidence indicating the relationship of anthropometric and hormonal measures with the characteristic morphology of polycystic ovary (PCO), defined as the presence of at least 12 follicles of 2–10 mm in one or both ovaries; in this definition is excluded the ovaries with a volume greater than 10 mL without cysts [35]. On analyzing the anthropometric variables of PCOS patients, BMI is significantly greater in PCOS patients with a characteristic PCO image than in those without that morphology. It has also been found that the hip perimeter is significantly greater in PCOS patients with characteristic image of PCO than in those without this ultrasonographic morphology [35]. In addition, it has been reported that PCOS patients with the PCO morphology by ultrasound display greater levels of testosterone than patients without it [35]. The probable explanation for greater testosterone levels when there is present the PCO morphology may be the bigger mass of altered ovarian tissue producing more steroids. Although the literature on this matter is scarce, an association between ovarian volume greater than 10 mL and total testosterone levels and LH/FSH ratio has been reported [36]. 

## 9. Adipocytokines 

Patients with PCOS—in comparison to control women— present lower serum levels of adiponectin but not of leptin. A decrease was observed in the expression of the ribonucleic acid (RNA) messenger of adiponectin in the subcutaneous and visceral adipose tissue, while that of leptin has been found significantly less only in the subcutaneous adipose tissue. Also, an inverse relationship between adiponectin and leptin expression as well as the measurement of subcutaneous and visceral adipose tissue by ultrasound has been observed [37]. Other authors have reported that obese PCOS but not normal weight PCOS patients have significantly lower adiponectin levels than control women [38]. Nonetheless, there are many difficulties with respect to the standardization for measure of these adipocytokines.

## 10. Metabolic Syndrome

The prevalence of metabolic syndrome is higher in PCOS patients than in control women (47% versus 4%, resp.) [39]. Free fatty acids, total cholesterol, and low-density lipoprotein cholesterol are higher in obese PCOS patients than in non obese PCOS patients [40]. Both PCOS and obesity are associated with dyslipidemia and endothelial dysfunction which increases the cardiovascular risk [41, 42]. There is evidence of a trend of deterioration in markers of endothelial dysfunction going from lean to obese PCOS women [41]. Although metabolic disorders usually prevail in the climacteric period, the risk of metabolic syndrome is high even at an early reproductive age [41]. 

Both PCOS and obesity induce an increase in serum inflammatory cardiovascular risk markers [43]. It has been reported an increased C-reactive protein, interleukin-6, and tumor necrosis factor alpha in obese PCOS patients with respect to control women; in addition, these markers have correlated with BMI and insulin resistance [43]. Non alcoholic fatty liver disease has been reported to be present in as high as 40% of PCOS patients associated to higher BMI, abdominal obesity, and worse lipid profile [44]. The pathogenic relationship among PCOS, obesity, metabolic, and cardiovascular disease is controversial. A low-grade chronic inflammation has been suggested as the potential cause of the long-term complications of PCOS [45]. 

## 11. Androgen Production in Obese and Non Obese PCOS Patients

**Figure 3:**
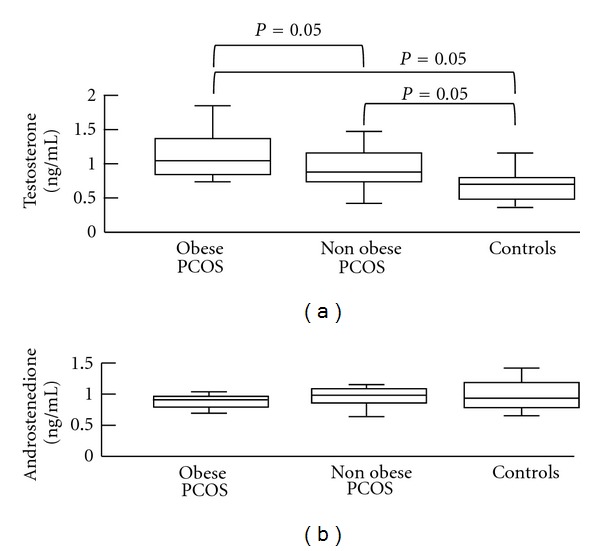
Values of total testosterone and androstenedione in obese and non obese patients with polycystic ovary syndrome and in control women. Box-and-whiskers plots of basal levels of androgens. The line within each box represents the median. Upper and lower boundaries of each box indicate 75th and 25th percentiles, respectively. The whiskers (above and below) show the upper and lower adjacent values, respectively. The levels of testosterone are significantly greater in the obese patients with PCOS compared with non obese PCOS patients and controls. Also, the testosterone levels are significantly greater in non obese PCOS patients than in control women. There are no significant differences in the levels of androstenedione. Modified from Moran et al. [48].

Controversy exists about the effect of obesity on serum androgen production in PCOS. Some investigators have reported that testosterone and androstenedione levels are similar in obese and non obese PCOS patients [23, 46]. However, it is well known that obesity generates a decrease in the sexual hormone-binding globulin, and therefore an increase in the levels of free androgens [22, 40]. Other studies have found that obesity generates an increase of testosterone levels in PCOS patients (Figure 3) [40, 47, 48]. In contrast, dynamic studies have shown lower androstenedione levels in obese PCOS patients than in non obese PCOS patients [33, 48].

Hyperandrogenism may be of ovarian or adrenal origin [49]. The adrenal participation in PCOS by the increment of dehydroepiandrosterone sulfate is found in 22–25% of PCOS patients [50]. However, some studies have found frequencies of hyperandrogenism due to dehydroepiandrosterone sulfate of 48–52% in different populations [51]. It has been reported that hyperandrogenic patients with higher adrenal androgen excess are leaner, younger and present more hirsutism than patients with lower levels of these same steroids [50]. 

## 12. Obesity in Pregnant PCOS Patients 

Since obesity and PCOS originate independently a deleterious effect on pregnancy and reproductive outcome, the impact of both conditions together is expectedly adverse in pregnant women and their fetuses. 

Obese patients with PCOS are characterized by a more severe hyperandrogenic and metabolic state, more irregular menses, less ovulatory cycles, and lower pregnancy rates, compared with normal weight PCOS patients. The importance of obesity in the pathogenesis of PCOS is evidenced by the efficacy of weight loss to improve metabolic alterations, to decrease hyperandrogenism, to increase ovulatory menstrual cycles, and to improve fertility [52]. The information with respect to the impact of obesity in hormonal and metabolic factors during intrauterine life is scarce as yet. 

## 13. Treatment of Obesity and Metabolic Abnormalities in PCOS Patients 

### 13.1. Modifications in Life Style 

The weight loss partially ameliorates hirsutism [53, 54], regularizes menstrual cycles and ovulation, as well as improves the endocrine and metabolic abnormalities [53–56]. 

### 13.2. Food Habit 

There is a known beneficial effect with the decrease of body weight and a worsening with the increase of excess weight in PCOS patients. It has been observed that some patients with PCOS can present menstrual cycles and ovulation after having reduced by only 5% of their body weight [54]. 

### 13.3. Exercise 

Physical activity has been found lower in PCOS patients than in control women [57]. The changes in lifestyle that incorporate an increase of physical activity and limited caloric intake have been beneficial in some studies. Regular physical activity is an important component to support the long-term reduction of overweight; however, the results are minimal with exercise alone [58]. An increase in physical activity is recommended for women with obesity and PCOS, as long as cardiovascular and orthopaedic limitations are taken into consideration [59]. 

### 13.4. Drugs for Obesity 

Drugs to control obesity have been used in obese patients with PCOS, although few studies exist to support this therapeutic approach. It is known that orlistat blocks the absorption of intestinal fat [60] and sibutramine suppresses the appetite [61]; both favour weight loss independently from the androgen excess and insulin resistance.  Nonetheless, it is important to take into account that these treatments must not be considered first-line treatments for obesity in patients with PCOS. 

### 13.5. Bariatric Surgery 

Few studies exist on the impact of bariatric weight loss surgery on manifestations of PCOS in patients with morbid obesity. The initial results of bariatric surgery in patients with morbid obesity and PCOS seem encouraging, since aside from the weight reduction many benefits are realized such as a decrease of hyperandrogenemia, hirsutism, and insulin resistance, and the restoration of menstrual cycles and ovulation is common [62].

## 14. Conclusion

The weight loss as an initial step of PCOS treatment is very important for overweight or obese PCOS patients, but not necessary for normal-weight PCOS patients, who only need to avoid increasing body weight. 

The differences in the hormonal and metabolic profile, as well as the different focus and response to treatment of obese and non obese PCOS patients, suggest that obesity has to be considered as a secondary characteristic for the classification of PCOS phenotypes. More prospective studies are needed to support this hypothesis. 

The intrauterine milieu in pregnancy and the reproduction outcome of PCOS patients with overweight or obesity are not clear. These topics are important for research in future prospective studies. 

PCOS and obesity induce an increase in serum inflammatory cardiovascular risk markers. The precise mechanisms underlying these associations require additional studies, to determine the relative contribution of different factors on cardiovascular disease. 

## Figures and Tables

**Table 1:** Phenotype classification of PCOS patients in different populations.

Features	Phenotype A HA + OA + PCO (%)	Phenotype B HA + OA (%)	Phenotype C HA + PCO (%)	Phenotype D OA + PCO (%)
Palermo, Italy^1^	53.9	8.9	28.8	8.4
Erzurum, Turkey^2^	44.1	22.8	14.2	18.9
Sao Paulo, Brazil^3^	58.4	7.9	11.1	22.6
Mexico City, Mexico^4^	64.5	32.6	2.9	0

HA: hyperandrogenism; OA: oligo-anovulation; PCO: polycystic ovaries. The NIH criteria recognizes A and B phenotypes, the Rotterdam consensus accepts all four phenotypes, and the AES-PCOS admits A, B, and C phenotypes. Data taken from ^1^Guastella et al. [14], ^2^Yilmaz et al. [15], ^3^Melo et al. [16], and ^4^Moran et al. [17].

**Table 2:** Phenotype classification in 172 patients with polycystic ovary syndrome taking into account obesity.

Features	A1 obese	A2 non obese	B1 obese	B2 non obese	C1 obese	C2 non obese
Hyperandrogenism	Yes	Yes	Yes	Yes	Yes	Yes
Oligo-anovulation	Yes	Yes	Yes	Yes	No	No
Polycystic ovaries	Yes	Yes	No	No	Yes	Yes
No. %	83 48.2	28 16.3	39 22.7	17 9.9	3 1.7	2 1.2

Obesity was considered evident when body mass index ≥27 and normal weight when BMI < 27. The frequencies of different phenotypes are taken from Moran et al. [17].

**Table 3:** Classification of hyperandrogenism in women.

Diagnosis	Mexico^1^ (*n* = 250) %	USA^2^ (*n* = 873) %	Italy^3^ (*n* = 950) %
Polycystic ovary syndrome	53.6	82.0	56.6
Idiopathic hirsutism/hyperandrogenism	24.8	4.5	7.6/15.8
Overweight or obesity^∗^	18.0	—	—
Hyperandrogenism and ovulation	—	6.7	15.5
Classic/nonclassic CAH	2.0	0.7/2.1	4.3
Androgen-secreting tumors	0.8	0.2	0.2
HAIRAN syndrome	—	3.8	—
Cushing's syndrome	0.4	—	—
Iatrogenic hirsutism	0.4	—	—

CAH: congenital adrenal hyperplasia. HAIRAN: hyperandrogenism, insulin resistance and acanthosis *nigricans*. ^∗^Hyperandrogenic patients with regular menstrual cycles. Taken from ^1^Moran et al. [7], ^2^Azziz et al. [8], ^3^Carmina et al. [9].

**Table 4:** Frequency of pathophysiologic components of polycystic ovary syndrome (PCOS).

Disorder	PCOS with obesity %	PCOS without obesity %	Total %
Gonadotropic dysfunction LH/FSH ≥ 2	19	25	22

Insulin resistance Insulin/glucose (pmol/mmol)	63^∗^	31^∗^	47

All the determinations were performed in one sample in fasting conditions. ^∗^Statistically significant difference (*P* < 0.01). From Moran et al. [24].
